# N-protein presents early in blood, dried blood and saliva during asymptomatic and symptomatic SARS-CoV-2 infection

**DOI:** 10.1038/s41467-021-22072-9

**Published:** 2021-03-26

**Authors:** Dandan Shan, Joseph M. Johnson, Syrena C. Fernandes, Hannah Suib, Soyoon Hwang, Danica Wuelfing, Muriel Mendes, Marcella Holdridge, Elaine M. Burke, Katie Beauregard, Ying Zhang, Megan Cleary, Samantha Xu, Xiao Yao, Purvish P. Patel, Tatiana Plavina, David H. Wilson, Lei Chang, Kim M. Kaiser, Jacob Nattermann, Susanne V. Schmidt, Eicke Latz, Kevin Hrusovsky, Dawn Mattoon, Andrew J. Ball

**Affiliations:** 1 Quanterix Corporation, Billerica, MA USA; 2 Institute of Innate Immunity, University of Bonn, Bonn, Germany; 3 German Center for Infection Research (DZIF), Bonn, Germany; 4 Department of Internal Medicine I, University of Bonn, Bonn, Germany; 5 German Center for Neurodegenerative Diseases (DZNE), Bonn, Germany

**Keywords:** SARS-CoV-2, Diagnostic markers, Viral infection

## Abstract

The COVID-19 pandemic continues to have an unprecedented impact on societies and economies worldwide. There remains an ongoing need for high-performance SARS-CoV-2 tests which may be broadly deployed for infection monitoring. Here we report a highly sensitive single molecule array (Simoa) immunoassay in development for detection of SARS-CoV-2 nucleocapsid protein (N-protein) in venous and capillary blood and saliva. In all matrices in the studies conducted to date we observe >98% negative percent agreement and >90% positive percent agreement with molecular testing for days 1–7 in symptomatic, asymptomatic, and pre-symptomatic PCR+ individuals. N-protein load decreases as anti-SARS-CoV-2 spike-IgG increases, and N-protein levels correlate with RT-PCR Ct-values in saliva, and between matched saliva and capillary blood samples. This Simoa SARS-CoV-2 N-protein assay effectively detects SARS-CoV-2 infection via measurement of antigen levels in blood or saliva, using non-invasive, swab-independent collection methods, offering potential for at home and point of care sample collection.

## Abstract (web summary)

Here the authors develop a single molecule array (Simoa) immunoassay for detection of SARS-CoV-2 nucleocapsid protein in venous and dried capillary blood as well as saliva. The assay shows good performance in symptomatic, asymptomatic, and pre-symptomatic PCR+ individuals.

## Introduction

At the end of 2019, the first cases of severe acute respiratory syndrome coronavirus-2 (SARS-CoV-2) infection were reported in Wuhan, China and the virus since has caused a worldwide pandemic^1^. Molecular testing for viral RNA is the primary diagnostic modality for active infection, while serological tests measure anti-SARS-CoV-2 antibodies post-infection^2, 3^. Although reverse transcriptase polymerase chain reaction (RT-PCR)-based molecular testing for viral RNA in respiratory specimens is the primary diagnostic tool for active infection, concerns have been raised about the risk of false-negative results associated with the use of nasal and nasopharyngeal (NP) swabs^4^, especially before symptom onset. Kucirka et al. estimate probability of a false-negative result to decrease from 100% on day 1 post-infection to 67% on day 4. On day 5, the median time for symptom onset, molecular tests still had a 38% probability of producing a false-negative result and declined no further than 20% in the days that followed, when the infection should be most detectable^5^. Furthermore, the complexity, significant supply chain challenges, and relatively low throughput of RT-PCR are contributing to the difficulties in developing sufficiently large-scale testing required to enable societies to re-open^6^, prompting a search for additional diagnostic modalities.

Antigen detection by immunoassay offers a simpler workflow and a supply chain diversified from PCR. Prior to January 2021, several SARS-CoV-2 antigen tests were approved by the US Food and Drug Administration (FDA) for use with NP or nasal swabs. These assays claim positive percent agreement (PPA) with PCR ranging from with 84 to 97.6%^7^. More recently, a single-molecule array (Simoa) SARS-CoV-2 N-Protein Antigen Test for NP swabs with 97.7% PPA received US FDA Emergency Use Authorization (EUA)^8^, indicating that highly sensitive and specific antigen testing is possible with this technology.

Detection of SARS-CoV-2 antigen in matrices beyond nasal and NP swabs may be of scientific and clinical significance^9^; indeed EUA applications have been authorized for molecular testing of SARS-CoV-2 in saliva, which allows for easier sample collection and may have better sensitivity than swab-based approaches^10^. Furthermore, multiple clinical manifestations suggest that this respiratory virus can migrate from the lungs into the bloodstream. Mehra et al. described evidence of SARS-CoV-2 peripheral involvement during post-mortem histological examination of effected tissues, including electron microscopic images of viral inclusion structures in endothelial cells^11^. It was hypothesized that SARS-CoV-2 infection may facilitate the induction of endothelitis in multiple organs as a direct consequence of viral involvement. Wölfel et al. reported that SARS-CoV-2 virus was not detectable in blood using molecular diagnostic techniques^12^, but additional later studies have found evidence that plasma viremia may play a significant role in disease course and that viral loads in plasma may predict risk of death^13–15^.

In this work, we describe development of a SARS-CoV-2 antigen test using Simoa technology to quantify N-protein in serum/plasma, dried blood microsamples (DBS), and saliva. The assay was designed to target the SARS-CoV-2 nucleocapsid protein, due to the large copy number per viral particle (~1000)^16^, and due to reports of large numbers of mutations in the SARS-CoV-2 spike protein^17^. We quantitate SARS-CoV-2 N-protein and anti-SARS-CoV-2 spike immunoglobulin G (IgG) directly in multiple sample matrices, including serum and plasma from venous collection, capillary blood acquired by finger-stick DBS devices (DBS), and saliva. Compared to molecular testing, we observe >90% PPA of SARS-CoV-2-positive patients and >98% negative percent agreement (NPA) in all matrices within 7 days of positive PCR test, both for asymptomatic and symptomatic patients, with the research assay described herein. An inverse relationship between N-protein and anti-SARS-CoV-2 spike protein IgG is observed, with antigen clearing as IgG increases. In longitudinal saliva and DBS samples, N-protein levels correlate between sample types and with Ct values measured in saliva. N-protein levels in saliva are higher but more variable than levels in capillary blood. The Simoa N-protein antigen test represents a robust SARS-CoV-2 detection tool in multiple types of sample matrix.

## Results

### Receiver operating characteristic curve (ROC) analysis and cutoff

We established preliminary cutoffs via ROC analysis for all sample types, as detailed in “Methods” in Supplementary Information. ROC curves are shown in Supplementary Fig. 1. Positive/negative cutoffs were determined to be the greater of either the functional limit of quantification (fLoQ) of the assay or the Youden Index recommended value. In all matrices, the cutoff was determined to be the fLoQ, as described in the assay data sheet (see “Methods”). The cutoff for SARS-CoV-2 spike IgG was as determined as per the EUA authorized test (see “Methods”). Dashed lines in figures represent the relevant positive/negative cutoff for each matrix. We consider these cutoffs as preliminary and acknowledge that they may change upon further studies.

### Serum and plasma samples

**Fig. 1:**
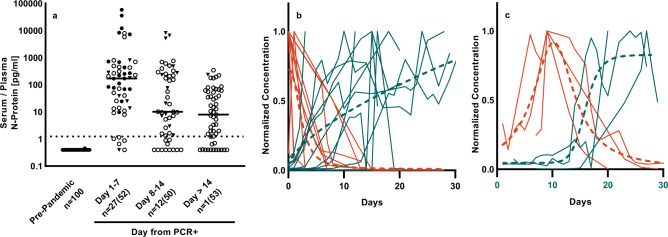
Simoa SARS-CoV-2 N-protein measurements differentiate pre-pandemic from PCR+ donors. **a** Shown are pre-pandemic sera and COVID-positive by molecular test (PCR+) plasma binned by day from PCR (BocaBio (triangles), U. Bonn (circles). First timepoint (closed symbol) and subsequent timepoints (open symbol) are shown. Data number is denoted as *n* = unique donors (total data points). Lines denote median value. **b** Concurrent decrease of N-protein (red) and increase of anti-SARS-CoV-2 spike IgG (blue). Time series with N-protein peak at day 1 (*n* = 10). **c** Time series with N-protein peak after day 1 (*n* = 3). Data were normalized to max and aligned at peak N-protein. Non-linear regression to the mean is shown in dashed lines. Non-normalized data are shown in Supplementary Fig. 2.

We measured N-protein in pre-pandemic sera (*n* = 100), in SARS-CoV-2 RT-PCR+ samples from a commercial source (*n* = 20) and in longitudinal plasma from the University of Bonn (*n* = 20 donors, total of *n* = 135 longitudinal samples). N-protein levels in each sample are shown binned by days from PCR in Fig. 1. With U. Bonn donors, PCR testing was performed and sample collection commenced on the first day of hospitalization; the date of symptom onset was not available for this cohort. Using a preliminary cutoff of 1.25 pg/mL N-protein (Fig. 1a, dashed line), the assay had an NPA of 100% and PPA of 97.5% with a molecular test in first-draw samples, independent of the number of days from PCR. Although most longitudinal samples remained positive for N-protein >14 days following initial PCR test (88% days 1–7, 78% days 8–14 and 72% day >14), a downward trend in N-protein concentrations over time was observed for individual donors.

Within the longitudinal cohort, we also measured anti-SARS-CoV-2 spike protein IgG for 141 timepoints over 16 patients (Supplementary Fig. 2). As N-protein decreased over time, we observed a concurrent increase in IgG. To explore the kinetics of N-protein levels relative to the serological response, we separated data sets into those with N-protein maximum concentration at day 1 (Fig. 1b; *n* = 10) and after day 1 (Fig. 1c; *n* = 3), normalized to maximum response and aligned to peak N-protein levels. Using non-linear regression to the mean, we determined that in these samples 3 days had elapsed between N-protein peak and seroconversion (Fig. 1c, from day 10 to day 13) and 10 days had elapsed between N-protein peak and IgG plateau (Fig. 1c, from day 10 to day 20). We defined seroconversion here as an increase to 5% of the max level measured. To investigate the possibility of post-seroconversion antigen masking influencing measured levels of N-protein, we treated longitudinal samples from patient 4 with dithiothreitol (DTT) to separate potential antigen–antibody complexes. We observed a negligible impact on N-protein levels, suggesting that antigen clearance, rather than antigen masking, causes the observed decrease in N-protein concentration (Supplementary Fig. 3).

### Dried blood microsamples

A total of 62 DBS samples were collected from 22 PCR+ and 15 PCR− individuals over multiple weeks in the presence of active COVID-19 infections from CTCH, a long-term care facility that established weekly testing of residents and staff using an FDA-authorized molecular test. An additional 64 PCR− samples were collected from a commercial source. Days of collection relative to initial PCR for the CTCH are shown in Table 1; full data is shown in Supplementary Information source data file on tab “CTCH Characteristics”.

**Table 1:** Sampling and testing timeline in the CTCH study.

Collection number	Collection 1	Collection 2	Collection 3
Relative day of PCR	1	8	No data
Relative day of DBS collection	5	12	29
Number of PCR+ donors	11	9	14
Number of PCR− donors	9	13	7
Notes	After collection 4 donors died, 1 declined, 7 new enrolled	After collection 1 donor died, 11 declined, 10 new enrolled	Follow-up timepoint that does not include recent PCR+ infections

**Fig. 2:**
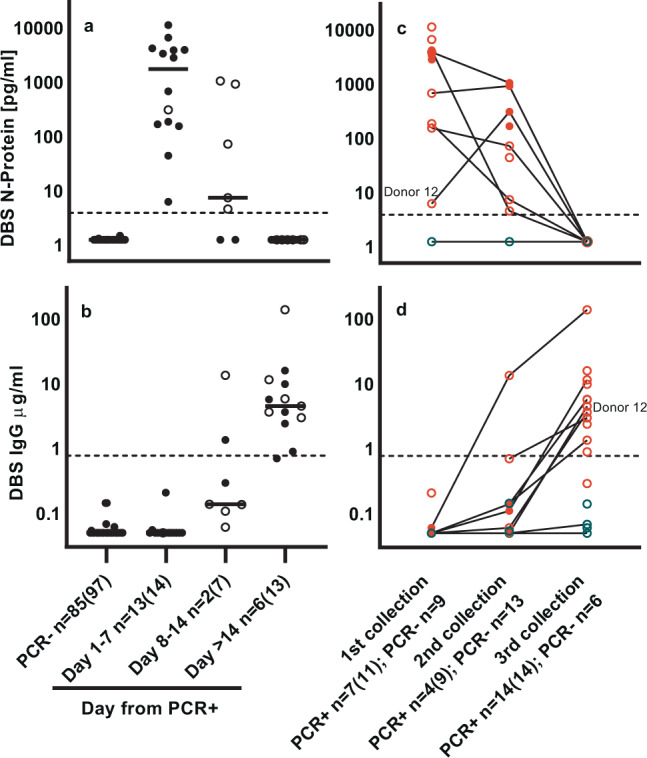
SARS-CoV-2 N-protein and anti-SARS-CoV-2 IgG in capillary blood (DBS) differentiate COVID PCR− from COVID PCR+ donors. **a** NP-protein in DBS. **b** IgG in DBS. Data are binned by day from PCR result. First timepoint (closed symbol) and subsequent timepoints (open symbol) are shown. Data number is denoted as *n* = unique donors (total data points). Lines denote median value. **c** N-protein over three collections. **d** IgG over three collections. Lines connect individual donors over multiple collections for PCR− (blue symbols), PCR+ with symptoms (red symbols), and PCR+ without symptoms (open red symbols) DBS. PCR+ *n* = donors without symptoms (total donors); PCR− *n* = total donors. Donor 12 is highlighted.

Figure 2a shows data binned by day from initial PCR test. Using a preliminary cutoff of 3.91 pg/mL, data demonstrate 100% NPA and PPA of the N-protein assay relative to RT-PCR for days 1–7. N-antigen is undetectable after 2 weeks post-PCR and IgG levels increase concomitantly. Figure 2b compares N-protein and IgG levels from individual donors over three collections (Table 1). In most donors, antigen monotonically decreases while IgG increases. A notable exception is Donor 12, a staff member who had N-protein levels above cutoff before developing symptoms or obtaining a positive PCR test, whose N-protein levels increased almost 50-fold by the time of second collection 1 week later. Nine donors had levels of N-protein above cutoff; some over multiple collections for a total 11 samples, despite an absence of SARS-CoV-2 symptoms (open red symbols). Anti-SARS-CoV-2 spike IgG levels increased above cutoff for 1 PCR+ donor by second collection and for 13 of the 14 donors at third collection.

**Fig. 3:**
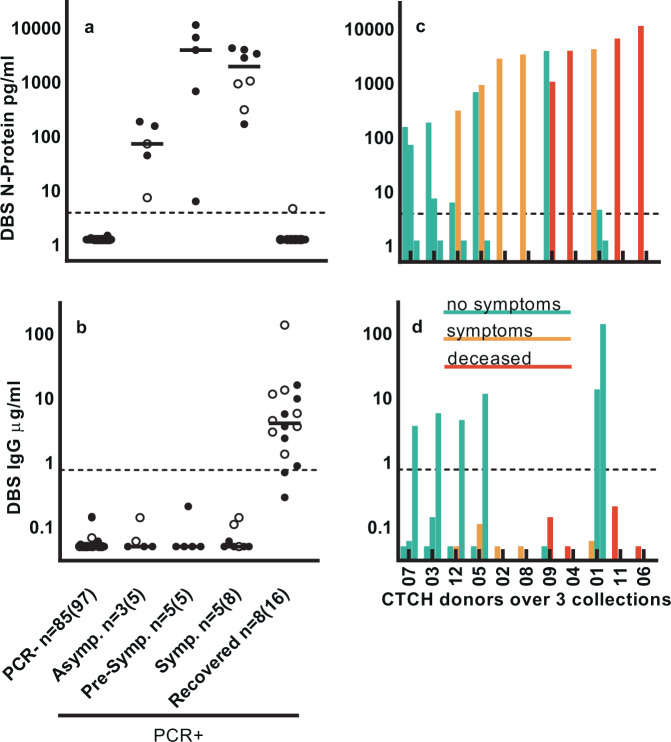
SARS-CoV-2 N-protein and anti-SARS-CoV-2 IgG levels in capillary blood (DBS) correlate with symptom severity indicators. **a** SARS-CoV-2 N-protein and **b** IgG concentrations segregated by symptom severity. First timepoint (closed symbol) and subsequent timepoints (open symbol) are shown. Data number is denoted as *n* = unique donors (total data points). Lines denote median value. **c** Ranking of donors with increasing N-protein levels, and color-coding associated with disease severity. Data are grouped by donors (*n* = 11), with each bar representing a single sample from 1 of the 3 collections (*n* = 11 sample collection 1; *n* = 6 sample collection 2; *n* = 5 sample collection 3). **d** IgG for same donors/collections as in **c**.

In Fig. 3, we present DBS data from these same donors, sorted by severity of reported symptoms. Figure 3a shows PCR− donors and PCR+ donors sorted into asymptomatic (donors with very mild or no symptoms reported throughout the study), pre-symptomatic (donors reported as symptom-free at initial collection but and reported with symptoms at a subsequent timepoint), symptomatic (including donors with symptoms reported at first test and pre-symptomatic donor timepoints after development of symptoms), and recovered (including novel donors >14 days after positive PCR test or with IgG above cutoff, pre-symptomatic and symptomatic donors after IgG increased above cutoff). Asymptomatic donors had a median level of N-protein of 72 pg/mL; median levels increased markedly in pre-symptomatic (3896 pg/mL) and symptomatic (1931 pg/mL) donors. Upon recovery from symptoms, N-protein mostly disappeared from the blood (7 or 8 donors below cutoff), decreases in N-protein being accompanied by a corresponding increase in IgG.

We ranked PCR+ donors by increasing N-protein level (from left to right) and color-coded results according to disease severity, defined from best to worst as: no symptoms (includes asymptomatic and pre-symptomatic), symptoms, and deceased (Fig. 3b). Worse disease severity associates with higher N-protein level: a two-sided Wilcoxon test showed a statistically significant difference in median N-protein levels between the no symptom (*n* = 5; 186.1 pg/mL) and symptom/deceased groups (*n* = 6; 4079.9 pg/mL) for the first collection (*p* = 0.0173). We observed concomitant IgG increase occurring for most donors at the third collection. Donor 1 was the only donor with N-protein >1000 pg/mL to recover and was the only donor to have anti-spike IgG levels above cutoff by the second collection, suggestive that the early IgG response was protective.

### Saliva

**Fig. 4:**
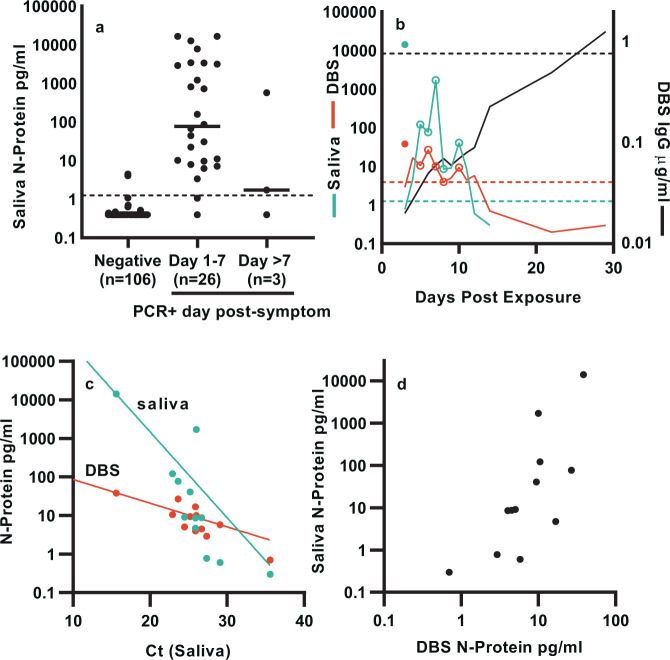
SARS-CoV-2 N-protein in saliva differentiate COVID PCR− from PCR+ donors and correlate with capillary blood (DBS). **a** N-protein concentration is binned by day from symptom onset. *n* = individual donors. **b** Matched saliva and DBS longitudinal samples from two donors. N-protein in saliva (blue line) and DBS (red line) and IgG in DBS (black line) for the index case (closed symbols) and housemate (lines). Open symbols represent days when symptoms are present for the housemate. **c** N-protein in matched saliva (blue symbols) and DBS (red symbols) as a function of cycle threshold (Ct) values, with exponential fits (solid lines). **d** Scatter plot of N-protein in saliva vs DBS.

Figure 4a shows the levels of N-protein for 25 pre-pandemic and 81 SARS-CoV-2 PCR− and 29 PCR+ saliva samples binned by days post-symptom. Applying a preliminary cutoff of 1.25 pg/mL to the saliva, data demonstrates 98.1% NPA and 92.3% PPA for the N-protein assay for days 1–7.

In a separate case study, we were able to use data from longitudinal, matched saliva and DBS samples to examine the kinetics and relative abundance of N-protein in these matrices over the course of an emerging infection from two donors that co-occupied a shared residence (Fig. 4b). The index case donor developed moderate SARS-CoV-2 symptoms and tested PCR positive by nasal swab on day 1. The housemate donor tested PCR negative on day 3 but developed mild symptoms on day 5. Matched saliva and DBS were positive for N-protein in the index case on the day of first antigen test (day 3) and the housemate 1 day before symptom onset (day 4). Daily sampling of the housemate revealed levels above cutoff until day 11 in saliva and day 12 in DBS. The housemate remained symptomatic from day 5 until day 8, with symptoms occurring on days with the highest levels of N-protein. Interestingly, symptoms resolved on day 9 after N-protein decreased, only to re-occur on day 10 along with a second N-protein peak. Anti-SARS-CoV-2 IgG monitored in DBS increased slowly, perhaps due to the mildness of symptoms, rising above cutoff on day 29.

We quantified RNA levels in the index case and housemate samples using a molecular test with EUA approval for saliva^18^. Figure 4c shows the correlation of Ct values of the N-gene RNA with N-protein in saliva and DBS, with correlation coefficient to the log2 transform of N-protein of −0.82 and −0.86, respectively. N-protein levels in saliva and DBS also correlate with a coefficient of 0.77 (Fig. 4d). In general, we observed higher and more variable levels of N-protein in saliva than in blood, although the overall distribution of levels is lower than observed in NP swabs (Supplementary Fig. 6).

## Discussion

We describe an ultrasensitive immunoassay that measures SARS-CoV-2 N-protein in venous blood, DBS, and saliva. In all matrices, we were able to detect N-protein in >90% of COVID-19 PCR+ donors, including those without symptoms. Although these data should not be considered as clinical validation, they strongly suggest that prospective clinical validation studies are merited.

In striking contrast to the high positivity levels of antigen in blood, SARS-CoV-2 RNAemia appears in a much lower percentage of patients than antigen, reported as ranging from 19.6 to 44%, though it correlates with worse disease outcome^13, 15, 19, 20^. This may be due to RNA being labile in circulation^21^. Ogata et al. also found that S1 antigen levels in blood correlate with worse disease outcome; however, they detected antigen (S1 or N-protein) in only 48 of the 64 patients with severe symptoms^22^. This may be due to assay differences, because >90% PPA of N-protein measurements in blood was observed for SARS^23^ and recently confirmed for SARS-CoV-2^24^.

Successful detection of SARS-CoV-2 antigen in DBS suggests potential feasibility of at-home collection. This method requires only 20 µL of capillary blood from a finger-stick, and specimens may be stored and shipped without cold-chain requirements. We report PPA >90% for DBS samples from day 1 to day 7 post PCR test. We observed lower levels of N-protein in DBS of asymptomatic compared to symptomatic patients; interestingly, we also observed a marked increase in pre-symptomatic DBS. This correlates with measurement showing the highest viral load in throat swabs before symptoms, from which peak infectiousness was also inferred to peak before symptom onset^25^. N-protein levels correlated with worse disease outcome in samples tested here, as has been observed for antigen previously^22^ and viral RNA as well^13^.

In saliva, also potentially suitable for home collection, we detected N-protein in >90% of COVID-19 PCR+ donors. When analyzing N-protein in longitudinal saliva and DBS samples from an infected donor, we observed that N-protein presented in both saliva and blood before symptom onset and that N-protein levels correlate with Ct values for RNA in saliva, as has been recently observed for N-protein in NP swabs^26^. Recent work suggests that viral load in saliva is a predictor of mortality^27^.

In all matrices, N-protein clearance was inversely correlated with an increase in SARS-CoV-2 anti-spike IgG. Seroconversion has been reported to occur between day 7 and day 13 post-symptom^28^; thus, based on our longitudinal data in plasma, we estimate N-protein peaks 4–10 days post-symptom, similar to timelines previously observed for SARS^23^ and SARS-CoV-2^22^. Our data preliminarily suggest that early IgG response alleviates severe disease outcome, even when high levels of N-protein are present.

False-negative PCR results have represented a significant challenge during the COVID-19 pandemic, particularly before onset of symptoms^4, 5, 29^. Compounding the problem of poor clinical discrimination in pre-symptomatic patients, He et al. observed the highest viral load in throat swabs at the time of symptom onset and inferred that infectiousness will peak at or before symptom onset^25^. In this context, the high PPA of the Simoa SARS-CoV-2 N-protein assay across multiple matrices may have utility to detect asymptomatic and pre-symptomatic individuals, although controlled clinical evaluation studies are required.

There are limitations to this work, particularly a limited availability of samples and incomplete clinical annotation for some samples. The U. Bonn and BocaBio cohorts tested (Fig. 1) were predominantly from hospitalized patients, reflecting N-protein levels from severe infection. Most CTCH samples (Figs. 2 and 3) were from residents predominantly of older age. We correlate saliva and DBS levels from only two donors (Fig. 4). We report PPA and NPA for the same retrospective samples in which we determined our cutoff, not on a separate or prospective cohort. The cutoffs described herein are preliminary and may change upon further investigation. In a separate but related study, a NIH-RADx-supported prospective sample collection is now ongoing, which will enable characterization of this Simoa N-protein test in a larger cohort of prospectively collected samples across multiple matrices. We also note that the HD-X instrument required for Simoa sample analysis is a laboratory-based instrument, therefore this Simoa N-protein test has associated instrument, set-up, and consumable costs. Use of automated laboratory instrumentation does provide throughput benefits; >1000 samples tested per 24-h period is possible per HD-X analyzer. Furthermore, many of the supply chain shortages that have limited molecular testing^30^ will not impact a Simoa blood/saliva test, since the Simoa test does not require transport media or swabs and does not rely on RNA extraction and amplification.

This study demonstrates that the Simoa SARS-CoV-2 N-protein assay readily detects viral antigen in active, pre-symptomatic, and asymptomatic COVID-19 infections in blood and saliva using sample collection methods that avoid swabs and the need to sample NP or nasal fluids. In addition to utility in studying the kinetics of SARS-CoV-2 infection, this assay may help expand the arsenal of SARS-CoV-2 antigen tests beyond nasal and NP swabs and enable blood- and/or saliva-based detection. Clinical validation studies are ongoing.

Simoa data shown herein was generated using research use only reagents, not in vitro diagnostic reagents or devices. The blood and saliva test described in this manuscript has not received an EUA and is not available in the United States for SARS-CoV-2 diagnostic uses.

## Methods

### Samples

Healthy pre-pandemic serum and plasma samples (collected before December 2019) were obtained from BiolVT (Westbury, NY). Commercially sourced serum and plasma samples from COVID-19-positive donors, as demonstrated by positive RT-PCR test, were obtained from Boca Biolistics (Pompano Beach, FL; hereafter “BocaBio”). Samples were collected between April 06 and June 17, 2020. RT-PCR was performed between March 06 and June 12, 2020. Plasma samples from hospitalized COVID-19 patients, as demonstrated by positive RT-PCR test, were provided by Dr. Jacob Nattermann, U. Bonn, Germany. Samples were collected between March 30 and April 22, 2020. RT-PCR was performed between March 30 and April 15, 2020. In COVID-19 patients who were not able to consent at the time of study enrollment, consent was obtained after recovery. DBS were collected using Mitra® Devices (Neoteryx, Torrance, CA) from staff and residents of Connecticut Baptist Care Homes Inc. (CTCH cohort). COVID-19 status of each donor was determined by RT-PCR test and DBS samples were collected at two timepoints, 1 week apart, for measurement of N-protein and IgG levels by Simoa. All staff and residents provided written informed consent prior to participating. Commercial saliva samples (pre-pandemic, PCR negative, and PCR positive) were sourced from Lee Biosolutions (Maryland Heights, MO). Matched DBS and saliva samples were (PCR positive) were collected from consented donors within Quanterix. Additional PCR− DBS and saliva samples were collected by Pharos Health (Baton Rouge, LA) from consented donors. The U. Bonn study was approved by the Institutional Review Board (IRB) of the University Hospital Bonn (134/20). All participants in other studies signed written informed consent prior to enrollment; samples were collected under an IRB exemption since these were fully de-identified samples.

### Inactivated virus

Gamma-inactivated SARS-CoV-2 virus was obtained from BEI; heat-inactivated SARS-CoV-2 and microbial specimens for cross-reactivity testing were obtained from ZeptoMetrix.

### Clinical characteristics

Symptoms from the CTCH cohort were reported by the facility director. Symptoms from the matched DBS–saliva donors (co-residents) were as self-reported. In all cases, symptoms were reported before data collection. Days from positive PCR are used in Figs. 1–3. Days from symptom onset were used in Fig. 4a and days from exposure used in Fig. 4b.

### Positive/negative cutoff

For N-protein cutoff, an ROC analysis of initial sample from single donors was performed for serum/plasma and saliva. For N-protein in DBS, multiple timepoints per donor were used due to a limited number of positive samples, and ROC analysis was confined to samples collected within 14 days of PCR. Any concentrations measured below the limit of detection (LOD) for each assay were replaced with the LOD (see “Assay development”). The Youden Index cutoff was compared to the fLoQ, and the positive/negative cutoff was chosen as the higher of the two. Cutoffs below fLoQ were avoided due to high variance, which may impact ability to assess positive and negative samples. Table 2 shows cutoff determination samples and statistics. ROC curves are shown in Supplementary Fig. 1 and statistics in the source data tabs SI Fig. 1A, SI Fig. 1B, and SI Fig. 1C. Multiple timepoints from individual donors were used for DBS ROC analysis, due to a limited number of positive samples.

**Table 2:** Cutoff determination for three matrices.

	Serum/plasma	DBS	Saliva
Negative *n*	100	97	106
Positive *n*	40	21	29
Youden Index	0.89	3.05	0.88
fLoQ	1.25	3.91	1.25
Cutoff chosen	1.25	3.91	1.25

The cutoffs for the N-protein assay are considered preliminary and may change upon further investigation.

The positive/negative cutoff for the IgG assay was determined during development of the Simoa SARS-CoV-2 spike IgG assay, and more information can be found in the Instructions for Use of the EUA (https://www.fda.gov/media/144764/download).

### Software and statistics

Data were collected using the Simoa HD-X analyzer using the Simoa HD-X software, version 3.0.2003.04001. Statistical analyses were performed using Graphpad prism (version 8.4.0 (671), Microsoft Excel (16.0.13530.20132), or R studio, R v.4.0.3 (package pROC)^31^. Non-linear regression to the mean (Fig. 1b, c) were done using either a Lorentzian (N-protein in Fig. 1C) or a 4PL (N-protein in Fig. 1b, IgG in Fig. 1b, c) logistic equations. To differentiate CTCH “no symptom” from “symptom groups” (collection 1), a two-sided Wilcoxon test was used to determine whether the median N-protein level on day 5 in asymptomatic patients was different from the median N-protein in symptomatic patients. The median measured N-protein levels on day 5 in symptomatic patients (4079.85) was higher than the median measured N-protein levels in asymptomatic patients (186.10) and Wilcoxon test showed that the difference was statistically significant (*p* = 0.0173). Correlations were calculated in Excel.

### Assay development

#### Simoa technology

Simoa technology offers analytical sensitivity on average 1000-fold greater than traditional immunoassay^32, 33^. In brief, the technology involves performing a paramagnetic microbead–based sandwich enzyme-linked immunosorbent assay, followed by isolation of individual capture beads in arrays of femtoliter-sized reaction wells. Singulation of capture beads within microwells permits buildup of fluorescent product from an enzyme label, so that signal from a single immunocomplex can be detected with a charge-coupled device camera in 30 s. At very low analyte concentrations, Poisson statistics dictate that bead-containing microwells in the array will contain either a single labeled analyte molecule or no analyte molecules, resulting in a digital signal of either “active” or “inactive” wells. Data collection involves counting active wells corresponding to single enzyme labels. At higher analyte concentrations, digital measurements transition to analog measurements of total fluorescence intensity. Simoa data are reported as average enzymes per bead. It is widely used in the field of neurodegenerative disease and, recently, for the measurement of SARS-CoV-2-associated biomarkers^34, 35^. It has also been demonstrated to rival the sensitivity of PCR for monitoring HIV infection through measurement of the p24 capsid protein in blood^36, 37^.

#### SARS-CoV-2 N-protein assay

Antibodies and antigens were obtained from commercial sources. Eight different antibodies and five antigens were screened, resulting in >60 different test configurations. The antibody and antigen combination that produced the best signal/background ratio for both calibrator and positive samples was selected. Diluent formulations, detector antibody, and Streptavidin–β-Galactosidase concentrations were then optimized, as well as assay protocols (two-step vs three-step; incubation times). A phosphate-based sample diluent was selected with EDTA to inhibit proteases, heterophilic blocker, and a detergent to help de-envelope and inactivate virus particles. For more information on assay performance and validation, including analytical LOD and LoQ, see https://www.quanterix.com/simoa-assay-kits/sars-cov-2-n-protein-antigen/. The Simoa® SARS CoV‐2 N-Protein Advantage Kit is commercially available through Quanterix Item #103806.

#### SARS-CoV-2 IgG assay

An assay was developed to monitor the serological response of IgG to the full spike of SARS-CoV-2. Details of the research use version of this assay can be found at https://www.quanterix.com/simoa-assay-kits/sars-cov-2-spike-igg/. The US FDA recently authorized the Simoa Semi-Quantitative SARS-CoV-2 IgG Antibody Test for Emergency Use—further details are available at https://www.fda.gov/media/144764/download. The Simoa® SARS-CoV-2 Spike IgG Advantage Kit is commercially available through Quanterix Item #103769.

### Sample types

Serum and plasma were collected by normal processing methods and stored frozen at −80 °C before analysis. Serum and plasma samples were diluted fourfold into assay diluent on the HD-X instrument before measurement. DBS were collected using Mitra collection kits from Neoteryx according to standard protocols (https://www.neoteryx.com/home-blood-blood-collection-kits-dried-capillary-blood). Tips absorb 20 µL of whole blood and are then allowed to dry for at least 16 h in a pouch with desiccant. Tips are extracted into 250 µL of assay diluent with shaking at 400 rpm overnight at 2–8 °C, resulting in a 12.5-fold sample dilution. No further on-board dilution is applied. Saliva samples were collected in polypropylene tubes without preservative and were stored frozen at −80 °C until the day of test. Saliva was clarified by centrifuging at 3000 × *g* for 10 min before testing, and diluted 4-fold on the HD-X. All sample results have been corrected for dilution factors, to represent the concentration within the sample matrix.

### Sample matrix correlation

To correlate serum and plasma matrices, matched samples from PCR+ donors were measured with the N-protein assay. N-protein levels correlated between matrices with a slope of 1.12 and an *R*^2^ of 0.995 (Supplementary Fig. 3). To verify the recovery of N-protein from the Mitra tips, whole blood was collected into K2EDTA tubes, spiked with recombinant N-protein, and then processed into either plasma or DBS. N-protein levels were measured in both sample types. N-protein levels correlate between matrices with *R*^2^ = 0.993 and a slope of 1.97. The concentration in DBS was approximately ½ of that in plasma, as expected due to the excluded volume of hematocrit, which is separated from plasma (Supplementary Fig. 4).

### DTT treatment of plasma samples

To determine whether seroconversion and antigen masking by Igs plays a role in the decrease of N-protein signal, samples were treated with 10 mM DTT at 37 °C for 15 min. To demonstrate the effectiveness of this treatment, the following experiment was conducted: (1) negative serum was spiked with N-protein and measured on the N-protein assay; (2) a 500× concentration of anti-N-protein antibody was added and the sample was measured, resulting in a 60% decrease in antigen; (3) the sample spiked with both antigen and antibody was treated with DTT according to the protocol above and measured, resulting in a 75% rescue of antigen signal (Supplementary Fig. 5).

### Cross-reactivity studies

Cultured and inactivated pathogens were spiked into negative serum samples to attain 10^5^ TCID50/mL, using a minimum of 4× dilution of viral stock into serum. Some virus cultures had insufficiently high stock titer to achieve 10^5^ TCID50/mL, and these viruses were tested at the highest titer possible after a 4× dilution into serum. No cross-reactivity was observed, as detailed in Supplementary Information Table 1.

### Reporting summary

Further information on research design is available in the Nature Research Reporting Summary linked to this article.

## Supplementary information

Supplementary Information

Reporting Summary

## Data Availability

The data that support the findings of this study are available within the manuscript and the supporting information. Source data are provided with this paper.

## Source data

Source Data

## References

[1] Roser, M. Ritchie, H., Ortiz-Ospina, E. & Hasell, J. Coronavirus pandemic (COVID-19). https://ourworldindata.org/coronavirus (2020).

[2] Amanat F (2020). A serological assay to detect SARS-CoV-2 seroconversion in humans. Nat. Med..

[3] Norman M (2020). Ultrasensitive high-resolution profiling of early seroconversion in patients with COVID-19. Nat. Biomed. Eng..

[4] Woloshin S, Patel N, Kesselheim AS (2020). False negative tests for SARS-CoV-2 infection - challenges and implications. N. Engl. J. Med..

[5] Kucirka LM, Lauer SA, Laeyendecker O, Boon D, Lessler J (2020). Variation in false-negative rate of reverse transcriptase polymerase chain reaction-based SARS-CoV-2 tests by time since exposure. Ann. Intern. Med..

[6] The COVID-19 testing debacle. *Nat. Biotechnol*. **38**, 653 (2020). 10.1038/s41587-020-0575-3 32493981

[7] U.S. Food & Drug Administration. Individual EUAs for antigen diagnostic tests for SARS-CoV-2. https://www.fda.gov/medical-devices/coronavirus-disease-2019-covid-19-emergency-use-authorizations-medical-devices/vitro-diagnostics-euas#individual-antigen (2020).

[8] US FDA. Simoa SARS-CoV-2 N Protein Antigen Test. Letter of authorization. https://www.fda.gov/media/144925/download (2021).

[9] Li L (2021). Analysis of viral load in different specimen types and serum antibody levels of COVID-19 patients. J. Transl. Med..

[10] Wyllie AL (2020). Saliva or nasopharyngeal swab specimens for detection of SARS-CoV-2. N. Engl. J. Med..

[11] Varga Z (2020). Endothelial cell infection and endotheliitis in COVID-19. Lancet.

[12] Wölfel R (2020). Virological assessment of hospitalized patients with COVID-2019. Nature.

[13] Fajnzylber, J. M. et al. SARS-CoV-2 viral load is associated with increased disease severity and mortality. *Nat. Commun*. **11**, 5493 (2020). 10.1038/s41467-020-19057-5 PMC7603483 33127906

[14] Di Cristanziano, V. et al. Detection of SARS-CoV-2 viremia before onset of COVID-19 symptoms in an allo-transplanted patient with acute leukemia. *Bone Marrow Transplant*. 10.1038/s41409-020-01059-y (2020). 10.1038/s41409-020-01059-y 32943755

[15] Eberhardt, K. A. et al. RNAemia corresponds to disease severity and antibody response in hospitalized COVID-19 patients. *Viruses* **12**, 1045 (2020). 10.3390/v12091045 PMC7551174 32962125

[16] Bar-On, Y. M., Flamholz, A., Phillips, R. & Milo, R. SARS-CoV-2 (COVID-19) by the numbers. *Elife* **9**, e57309 (2020). 10.7554/eLife.57309 PMC7224694 32228860

[17] Ou, J. et al. Emergence of SARS-CoV-2 spike RBD mutants that enhance viral infectivity through increased human ACE2 receptor binding affinity. Preprint at *bioRxiv*10.1101/2020.03.15.991844 (2020).

[18] Infinity BiologiX LLC. Infinity BiologiX TaqPath SARS-CoV-2 assay. Molecular IFU https://www.fda.gov/media/137773/download (2020).

[19] Hogan, C. A. et al. High frequency of SARS-CoV-2 RNAemia and association with severe disease. *Clin. Infect. Dis*. 10.1093/cid/ciaa1054 (2020). 10.1093/cid/ciaa1054 PMC7543277 32965474

[20] Kawasuji, H. et al. SARS-CoV-2 RNAemia with higher nasopharyngeal viral load is strongly associated with severity and mortality in patients with COVID-19. Preprint at *medRxiv*10.1101/2020.12.17.20248388 (2020). 10.1002/jmv.27282 PMC8426802 34411312

[21] Tsui NBY, Ng EKO, Lo YMD (2002). Stability of endogenous and added RNA in blood specimens, serum, and plasma. Clin. Chem..

[22] Ogata AF (2020). Ultra-sensitive serial profiling of SARS-CoV-2 antigens and antibodies in plasma to understand disease progression in COVID-19 patients with severe disease. Clin. Chem..

[23] Che X-Y (2004). Nucleocapsid protein as early diagnostic marker for SARS. Emerg. Infect. Dis..

[24] Hingrat, Q. L. E. et al. Detection of SARS-CoV-2 N-antigen in blood during acute COVID-19 provides a sensitive new marker and new testing alternatives. *Clin. Microbiol. Infect*. 10.1016/j.cmi.2020.11.025 (2020). 10.1016/j.cmi.2020.11.025 PMC7724284 33307227

[25] He X (2020). Temporal dynamics in viral shedding and transmissibility of COVID-19. Nat. Med..

[26] Pollock, N. R. et al. Correlation of SARS-CoV-2 nucleocapsid antigen and RNA concentrations in nasopharyngeal samples from children and adults using an ultrasensitive and quantitative antigen assay. *J. Clin. Microbiol.* https://doi.org/10.1128/JCM.03077-20 (2021). 10.1128/JCM.03077-20 PMC8092747 33441395

[27] Silva, J. et al. Saliva viral load is a dynamic unifying correlate of COVID-19 severity and mortality. Preprint at *medRxiv*10.1101/2021.01.04.21249236 (2021).

[28] Long Q-X (2020). Antibody responses to SARS-CoV-2 in patients with COVID-19. Nat. Med..

[29] Lauer SA (2020). The incubation period of coronavirus disease 2019 (COVID-19) from publicly reported confirmed cases: estimation and application. Ann. Intern. Med..

[30] American Society for Microbiology. Supply shortages impacting COVID-19 and non-COVID testing. https://asm.org/Articles/2020/September/Clinical-Microbiology-Supply-Shortage-Collecti-1 (2020).

[31] Robin X (2011). pROC: an open-source package for R and S+ to analyze and compare ROC curves. BMC Bioinformatics.

[32] Rissin DM (2010). Single-molecule enzyme-linked immunosorbent assay detects serum proteins at subfemtomolar concentrations. Nat. Biotechnol..

[33] Wilson DH (2016). The Simoa HD-1 analyzer: a novel fully automated digital immunoassay analyzer with single-molecule sensitivity and multiplexing. J. Lab. Autom..

[34] Kanberg N (2020). Neurochemical evidence of astrocytic and neuronal injury commonly found in COVID-19. Neurology.

[35] Ameres M (2020). Association of neuronal injury blood marker neurofilament light chain with mild-to-moderate COVID-19. J. Neurol..

[36] Chang L (2013). Simple diffusion-constrained immunoassay for p24 protein with the sensitivity of nucleic acid amplification for detecting acute HIV infection. J. Virol. Methods.

[37] Cabrera C, Chang L, Stone M, Busch M, Wilson DH (2015). Rapid, fully automated digital immunoassay for p24 protein with the sensitivity of nucleic acid amplification for detecting acute HIV infection. Clin. Chem..

