# Supplementary material for: N-protein presents early in blood, dried blood and saliva during asymptomatic and symptomatic SARS-CoV-2 infection
Source: Nat Commun. 2021 Mar 26;12:1931. doi: 10.1038/s41467-021-22072-9 (PMC7997897; doi:10.1038/s41467-021-22072-9)
Supplement: Supplementary file 1 — Supplementary Information [file 41467_2021_22072_MOESM1_ESM.pdf]

Supplementary Information.

N-protein presents early in blood, dried blood and saliva during asymptomatic and symptomatic SARS-CoV-2 infection.

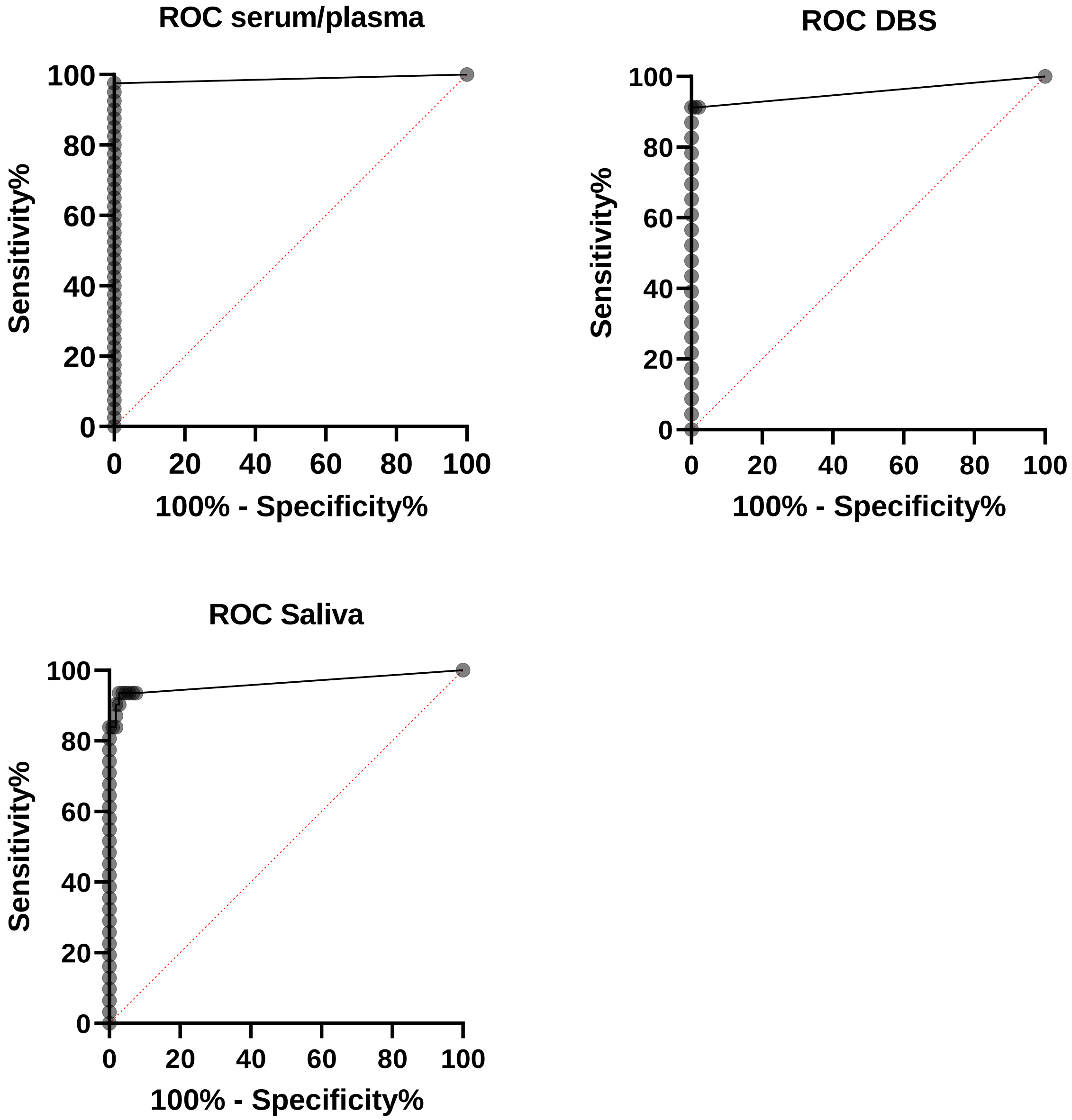

Supplementary Figure 1 – ROC curves for serum/plasma, DBS (within 14 days of positive PCR) and saliva. See Positive / Negative Cutoff in Methods for more information.

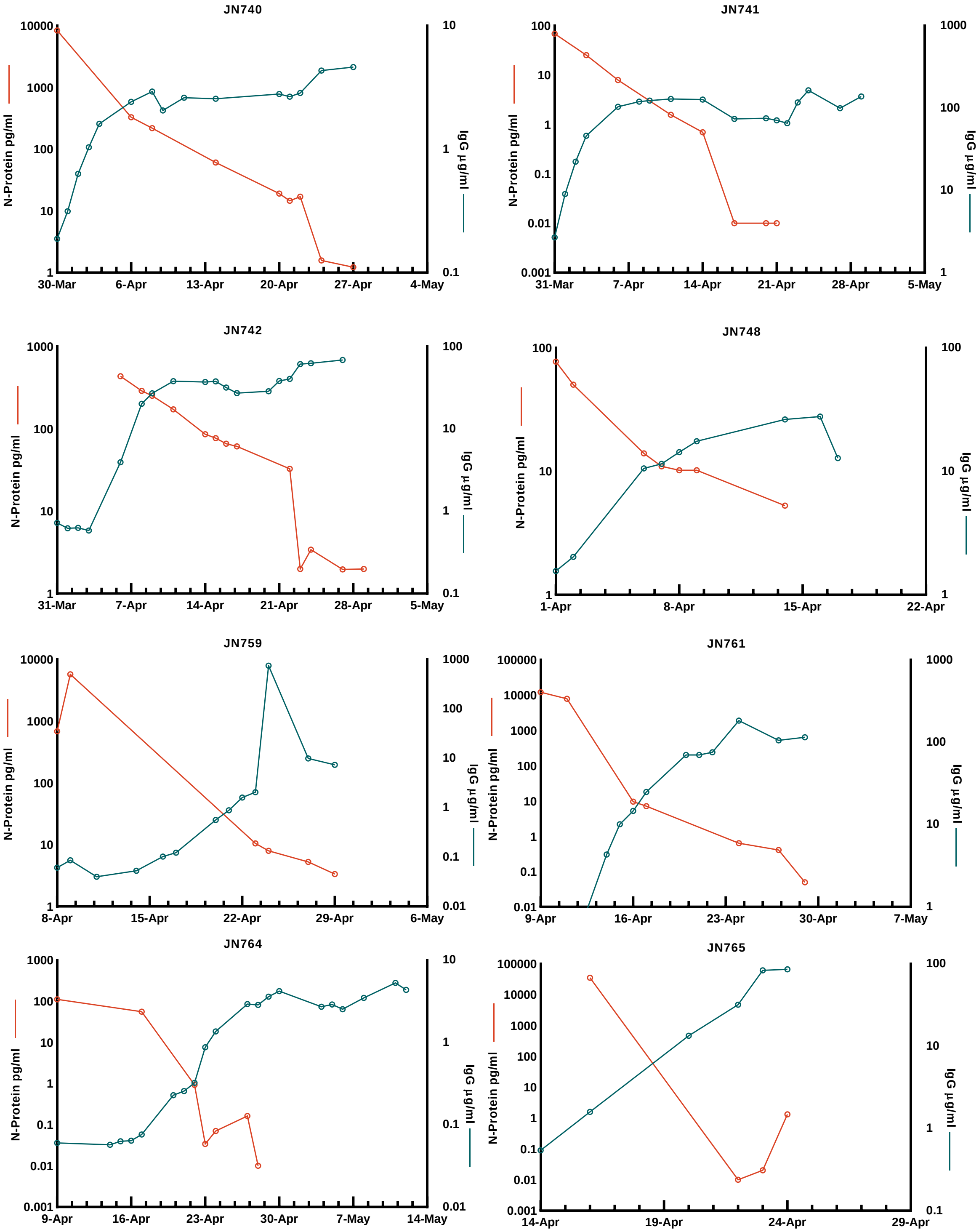
JN740
10000
1000
100
10
1
N-Protein pg/ml
10
1
0.1
IgG μg/ml
30-Mar
6-Apr
13-Apr
20-Apr
27-Apr
4-May
JN741
100
10
1
0.1
0.01
0.001
N-Protein pg/ml
1000
100
10
1
IgG μg/ml
31-Mar
7-Apr
14-Apr
21-Apr
28-Apr
5-May
JN742
1000
100
10
1
N-Protein pg/ml
100
10
1
0.1
IgG μg/ml
31-Mar
7-Apr
14-Apr
21-Apr
28-Apr
5-May
JN748
100
10
1
N-Protein pg/ml
100
10
1
IgG μg/ml
1-Apr
8-Apr
15-Apr
22-Apr
JN759
10000
1000
100
10
1
N-Protein pg/ml
1000
100
10
1
0.1
0.01
IgG μg/ml
8-Apr
15-Apr
22-Apr
29-Apr
6-May
JN761
100000
10000
1000
100
10
1
0.1
0.01
N-Protein pg/ml
1000
100
10
1
IgG μg/ml
9-Apr
16-Apr
23-Apr
30-Apr
7-May
JN764
1000
100
10
1
0.1
0.01
0.001
N-Protein pg/ml
10
1
0.1
0.01
IgG μg/ml
9-Apr
16-Apr
23-Apr
30-Apr
7-May
14-May
JN765
100000
10000
1000
100
10
1
0.1
0.01
0.001
N-Protein pg/ml
100
10
1
0.1
IgG μg/ml
14-Apr
19-Apr
24-Apr
29-Apr

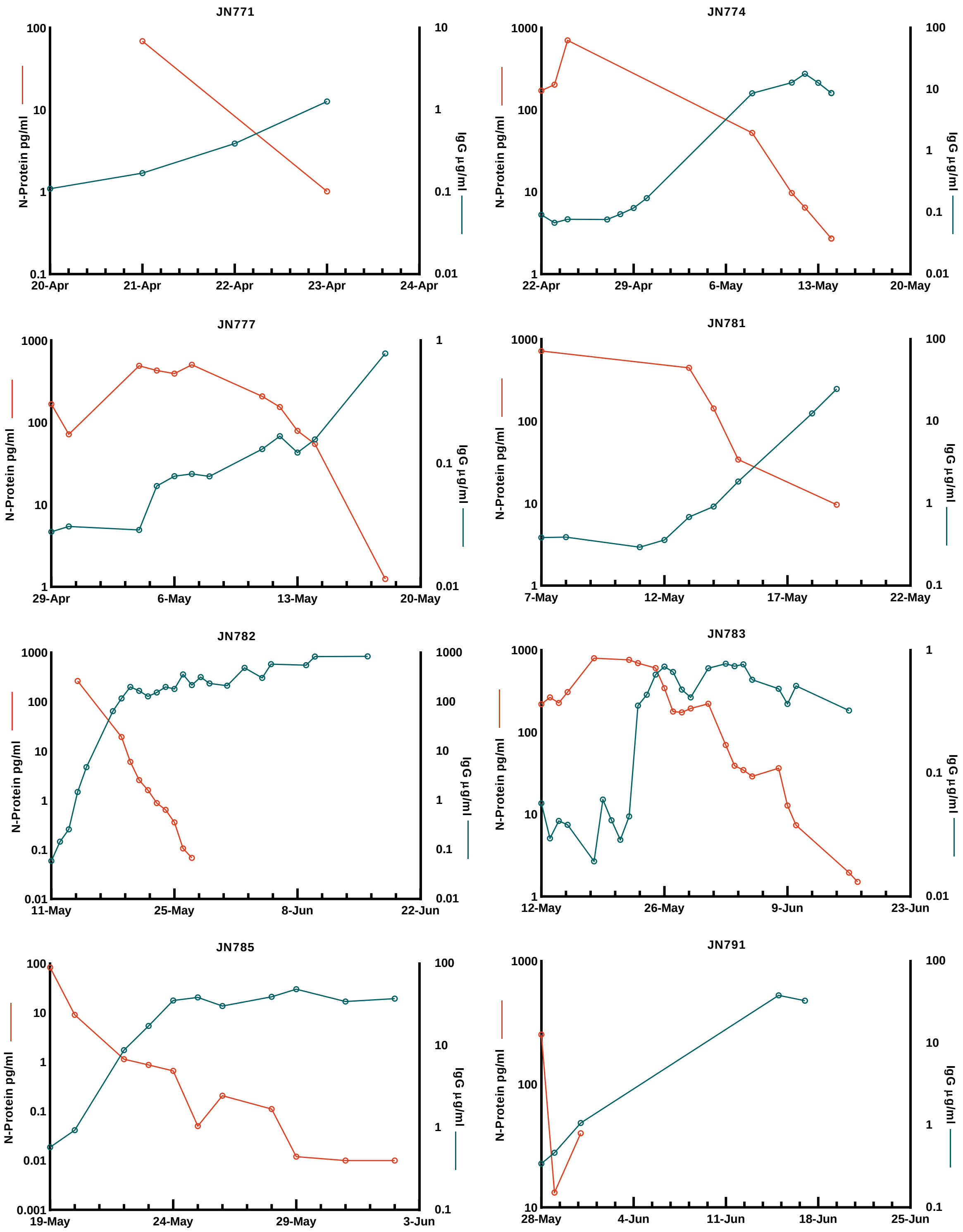

Supplementary Figure 2 – SARS-CoV-2 N-protein and anti-spike IgG levels in plasma from the Univ of Bonn cohort. Sixteen individual donors were sampled over multiple timepoints for a total of 141 data points. N-Protein is shown in red, and IgG in blue.

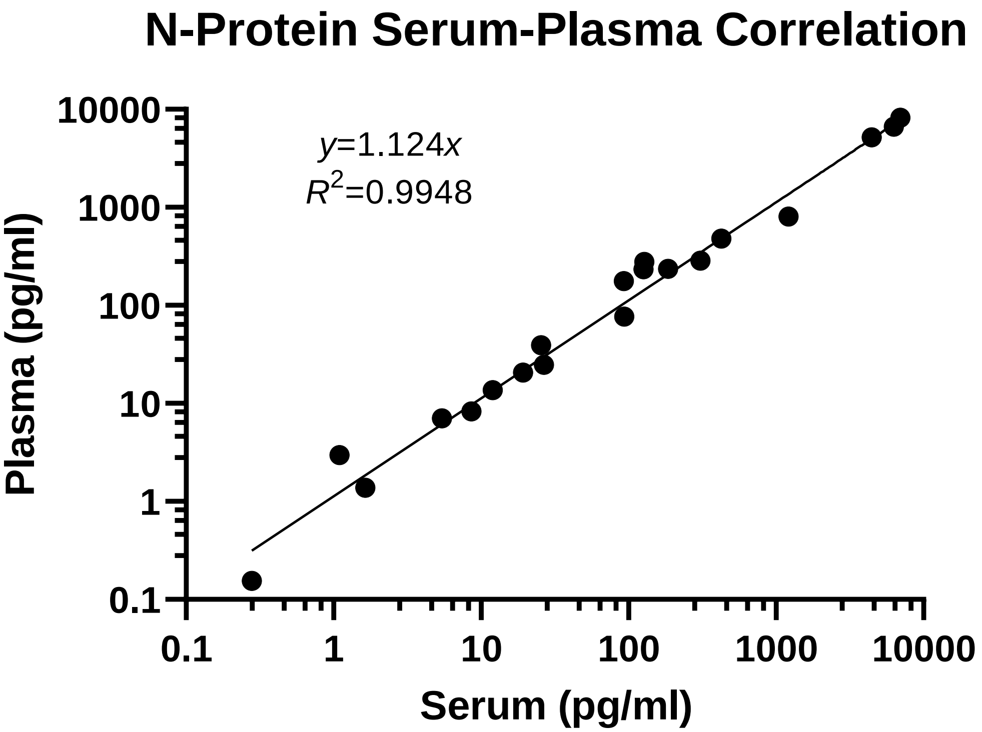

Supplementary Figure 3. Matched serum and plasma samples from the same donors were found to have correlation in N antigen levels between matrices. Twenty matched samples from BocaBio confirmed to be PCR+ were tested in both serum and K2 EDTA plasma.

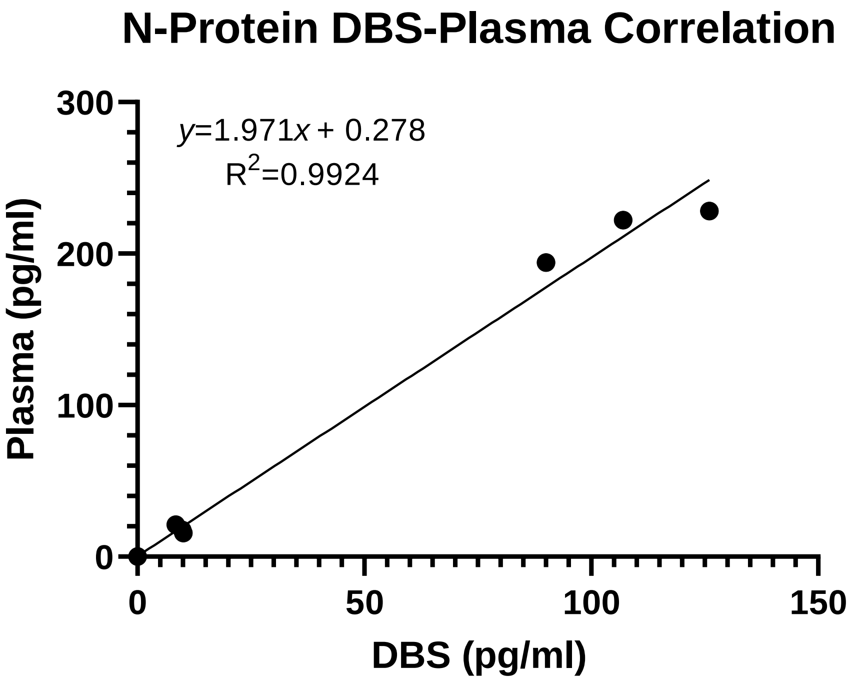

Supplementary Figure 4. Whole blood drawn into a K2 EDTA plasma tube (3 donors) was spiked with known levels of recombinant N-protein. It was then processed into neat plasma and in parallel into Dried Blood Microsamples (DBS) using Neoteryx Mitra tips. After extraction, both sample types were measured, showing a correlation of 0.99. The concentration in DBS was approximately ½ of that in plasma, as expected due to the excluded volume of hematocrit which is separated from plasma.

A.

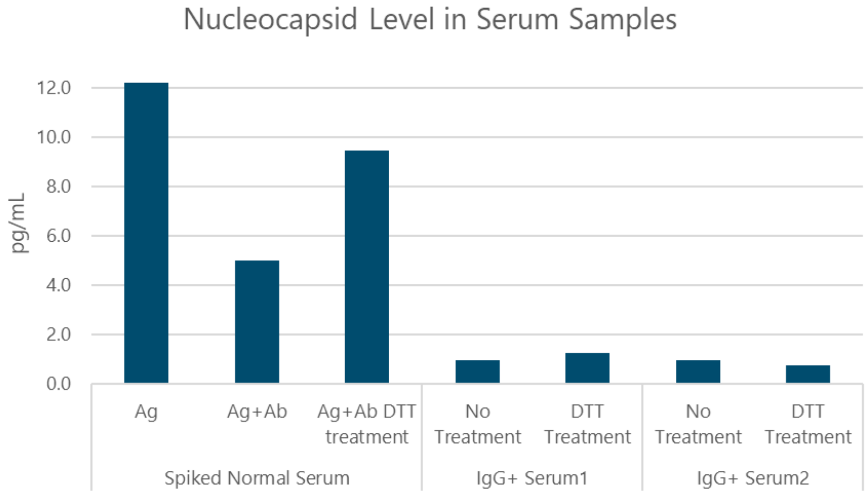

B.

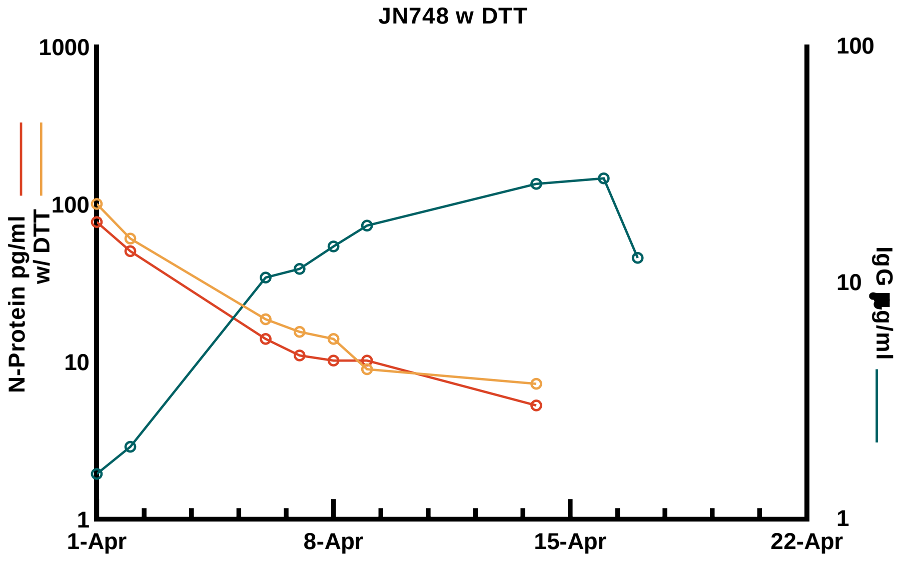

Supplementary Figure 5. Panel A. A DTT reduction protocol was established to unmask N-protein bound by antibody in serum by doing a control experiment with recombinant antigen and capture antibody spiked into sample matrix. N-protein concentration measured in serum was reduced after co-spiking with antibody, indicative of epitope masking. Adding DTT to the sample rescued 63% of the signal loss, indicating that this treatment could unmask antigen in seroconverted samples. **Panel B.** Longitudinal plasma samples from Donor 4 Univ of Bonn, treated with and without DTT before testing. Samples were not treated with DTT before testing for IgG. Negligible decrease in N-protein levels were observed with DTT treatment, suggesting that antibody-antigen complexes were not masking N-protein signal, or causing the observed decrease over time. N-Protein is shown in red, N-Protein from samples treated with DTT is shown in orange, and IgG is shown in blue.

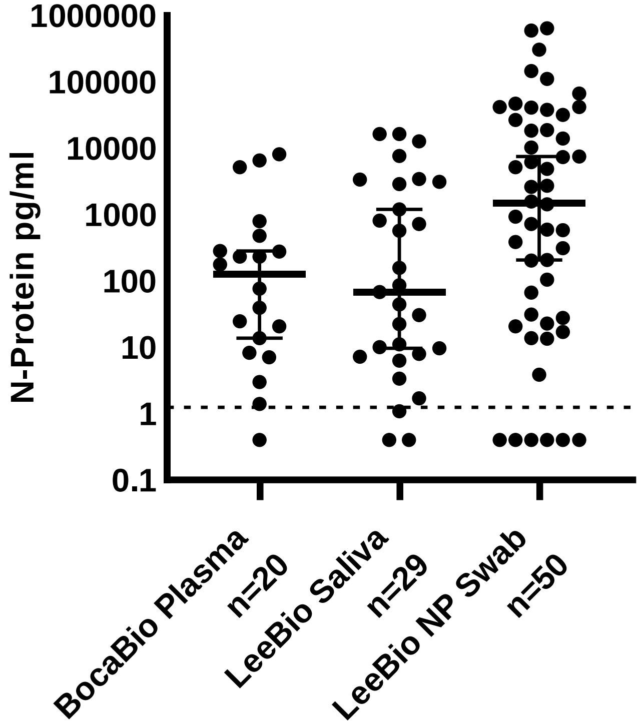

Supplementary Figure 6. N-protein measured in plasma, saliva and nasopharyngeal swabs from commercial sources. The solid lines represent median values (+/- 95% confidence interval).

Table S1. Inactivated, cultured virus was purchased from Zeptometrix, and tested for cross-reactivity at the $TCID_{50}$ levels listed. No cross-reactivity was observed.

Virus Description	Vendor	Cat#	Titer Tested $TCID_{50}$/mL	Conc Measured by N antigen Assay	
				Serum	Plasma
Adenovirus Type 07 (Species B) Culture Fluid	Zeptometrix	0810021CF HI	3.52E+04	<LoD	<LoD
Enterovirus Type 68 (2007 Isolate) Culture Fluid	Zeptometrix	0810237CF HI	3.78E+05	<LoD	<LoD
Influenza A H1N1 (New Cal/20/99) Culture Fluid	Zeptometrix	0810036CF HI	2.88E+05	<LoD	<LoD
Influenza B (Florida/02/06) Culture Fluid	Zeptometrix	0810037CF HI	3.52E+04	<LoD	<LoD
Parainfluenza Virus Type 1 Culture Fluid	Zeptometrix	0810014CF HI	2.28E+06	<LoD	<LoD
Parainfluenza Virus Type 2 Culture Fluid	Zeptometrix	0810015CF HI	2.88E+05	<LoD	<LoD
Parainfluenza Virus Type 3 Culture Fluid	Zeptometrix	0810016CF HI	1.65E+06	<LoD	<LoD
Parainfluenza Virus Type 4A Culture Fluid	Zeptometrix	0810060CF HI	7.05E+05	<LoD	<LoD
Respiratory Syncytial Virus Type A (Isolate: 2006 Isolate) Culture Fluid	Zeptometrix	0810040AC FHI	9.50E+05	<LoD	<LoD
Rhinovirus Type 1A Culture Fluid	Zeptometrix	0810012CF NHI	8.88E+04	<LoD	<LoD
Coronavirus (Strain: 229E) Culture Fluid	Zeptometrix	0810229CF HI	1.04E+05	<LoD	<LoD
Coronavirus (Strain: OC43) Culture Fluid	Zeptometrix	0810024CF HI	2.63E+05	<LoD	<LoD
Coronavirus (Strain: NL63) Culture Fluid	Zeptometrix	0810228CF HI	4.25E+04	<LoD	<LoD
