# Supplementary material for: N-protein presents early in blood, dried blood and saliva during asymptomatic and symptomatic SARS-CoV-2 infection
Source: Nat Commun. 2021 Mar 26;12:1931. doi: 10.1038/s41467-021-22072-9 (PMC7997897; doi:10.1038/s41467-021-22072-9)
Supplement: Supplementary file 2 — Reporting Summary [file 41467_2021_22072_MOESM2_ESM.pdf]

nature research

Corresponding author(s): Andrew J. Ball
Last updated by author(s): Feb 11, 2021

Reporting Summary

Nature Research wishes to improve the reproducibility of the work that we publish. This form provides structure for consistency and transparency in reporting. For further information on Nature Research policies, see our Editorial Policies and the Editorial Policy Checklist.

Statistics

For all statistical analyses, confirm that the following items are present in the figure legend, table legend, main text, or Methods section.

n/a	Confirmed	
☐	☒	The exact sample size (*n*) for each experimental group/condition, given as a discrete number and unit of measurement
☐	☒	A statement on whether measurements were taken from distinct samples or whether the same sample was measured repeatedly
☐	☒	The statistical test(s) used AND whether they are one- or two-sided<br>*Only common tests should be described solely by name; describe more complex techniques in the Methods section.*
☒	☐	A description of all covariates tested
☐	☒	A description of any assumptions or corrections, such as tests of normality and adjustment for multiple comparisons
☐	☒	A full description of the statistical parameters including central tendency (e.g. means) or other basic estimates (e.g. regression coefficient) AND variation (e.g. standard deviation) or associated estimates of uncertainty (e.g. confidence intervals)
☐	☒	For null hypothesis testing, the test statistic (e.g. *F*, *t*, *r*) with confidence intervals, effect sizes, degrees of freedom and *P* value noted<br>*Give P values as exact values whenever suitable.*
☒	☐	For Bayesian analysis, information on the choice of priors and Markov chain Monte Carlo settings
☒	☐	For hierarchical and complex designs, identification of the appropriate level for tests and full reporting of outcomes
☒	☐	Estimates of effect sizes (e.g. Cohen's *d*, Pearson's *r*), indicating how they were calculated

Our web collection on statistics for biologists contains articles on many of the points above.

Software and code

Policy information about availability of computer code

Data collection	Commercially available Simoa HD-X software, version 3.0.2003.04001
Data analysis	Commercially available Graphpad prism (version 8.4.0 (671), Microsoft Excel 45 (16.0.13530.20132), and open source R studio, R v.4.0.3 (package pROC)1

For manuscripts utilizing custom algorithms or software that are central to the research but not yet described in published literature, software must be made available to editors and reviewers. We strongly encourage code deposition in a community repository (e.g. GitHub). See the Nature Research guidelines for submitting code & software for further information.

Data

Policy information about availability of data

All manuscripts must include a data availability statement. This statement should provide the following information, where applicable:

- Accession codes, unique identifiers, or web links for publicly available datasets
- A list of figures that have associated raw data
- A description of any restrictions on data availability

Data availability statement is included in the manuscript. The data that support the findings of this study are available from the corresponding author upon reasonable request. Data for all figures in the main and supplementary text, as well as clinical information for the CTCH cohort are contained within the Source Data excel file provided to the journal. No data meeting the descriptions of mandatory deposition datasets are included in this manuscript.

Field-specific reporting

Please select the one below that is the best fit for your research. If you are not sure, read the appropriate sections before making your selection.

☒ Life sciences ☐ Behavioural & social sciences ☐ Ecological, evolutionary & environmental sciences

For a reference copy of the document with all sections, see nature.com/documents/nr-reporting-summary-flat.pdf

Life sciences study design

All studies must disclose on these points even when the disclosure is negative.

Sample size	Sample size was primarily determined by sample availability. This is stated in the limitations paragraph:<br>There are limitations to this work, particularly a limited availability of samples and incomplete clinical annotation for some samples. The JN and BocaBio cohorts tested (Figure 1) were predominantly from hospitalized patients, reflecting N-Protein levels from severe infection. Most CTCH samples (Fig 2 & 3) were from residents predominantly of older age. We correlate saliva and DBS levels from only two donors (Figure 4). We report PPA and NPA for the same retrospective samples in which we determined our cutoff, not on a separate or prospective cohort. The cutoffs described herein are preliminary and may change upon further investigation. In a separate but related study, a NIH-RADx supported prospective sample collection is now ongoing, which will enable characterization of this Simoa N-protein test in a larger cohort of prospectively collected samples across multiple matrices.
Data exclusions	No data were excluded from analysis.
Replication	Upon development/selection of optimal assay conditions for the tests described in the manuscript, all attempts at replication were successful. All assay runs included at least two control samples with known levels of analyte - all runs in this study reported concentration of these control samples within acceptable ranges. Within this study, N-protein and anti-spike IgG were measured in multiple cohorts over multiple runs. A select set of samples were tested on at least two runs, returning results <20% CV on average.
Randomization	Samples were not randomized. Samples were organized into COVID positive or negative groups depending upon the comparator molecular test, unless PCR status was unknown.
Blinding	The majority of samples tested were retrospectively collected from commercial sources or collaborators, with a view to determining the ability of the tests described here to detect SARS-CoV-2 antigen and/or IgG status. These samples reached the operators with their PCR or SARS-CoV-2 IgG status already known, thus operators were not always blinded to SARS-CoV-2 infection status of samples being tested. Exceptions to this were the CTCH DBS cohort and the longitudinal housemate matched saliva-DBS samples, which were of unknown PCR status before testing. We recognize that ideally all samples would have been blinded from the operators during all testing. Subsequent clinical evaluation studies with prospectively collected cohorts were run blinded, however those data was not in scope for this publication, which focuses on initial development and characterization of the assays.

Reporting for specific materials, systems and methods

We require information from authors about some types of materials, experimental systems and methods used in many studies. Here, indicate whether each material, system or method listed is relevant to your study. If you are not sure if a list item applies to your research, read the appropriate section before selecting a response.

Materials & experimental systems

n/a	Involved in the study	
☐	☒	Antibodies
☒	☐	Eukaryotic cell lines
☒	☐	Palaeontology and archaeology
☒	☐	Animals and other organisms
☐	☒	Human research participants
☒	☐	Clinical data
☒	☐	Dual use research of concern

Methods

n/a	Involved in the study	
☒	☐	ChIP-seq
☒	☐	Flow cytometry
☒	☐	MRI-based neuroimaging

Antibodies

Antibodies used	This article describes a commercial product, and the components of that product including antibodies are considered trade secret. Upon reasonable request, assay kits can be made available to reproduce the work described herein. It is our general practice to not reveal antibody information publically. Nonetheless, in the interest of transparency, the antibodies used in the test described here were:<br><br>N protein assay<br>Supplier name: Sino Biological<br>Catalog number: 40143-MM05

Species / Isotype: Mouse Monoclonal IgG1
Clone name: 05
Lot number/s: MA14JU0802

Supplier name: Sino Biological
Catalog number: 40143-R004
Species / Isotype: Rabbit Monoclonal IgG
Clone name: 004
Lot number/s: MA14JU1901, HA14AP0701

Spike-IgG assay
Supplier name: Bethyl Laboratories
Catalog number: A80-148B
Species / Isotype: Goat Polyclonal F(ab')2
Clone name: n/a
Lot number/s: A80-148B-4

Validation

Validation testing performed by antibody vendors was as follows:

Supplier name: Sino Biological
Catalog number: 40143-MM05
Antibody validation by vendor: Cross-reactivity in ELISA and Western Blot with SARS-CoV-2 (2019-nCoV) Nucleocapsid Protein. No cross-reactivity in ELISA with MERS-CoV Nucleoprotein protein, HCoV-229E Nucleoprotein protein, HCoV-NL63 Nucleoprotein protein, HCoV-HKU1Nucleoprotein protein, HCoV-OC43 Nucleoprotein.

Supplier name: Sino Biological
Catalog number: 40143-R004
Antibody validation by vendor: Cross-reactivity in ELISA and WB with SARS-CoV-2 (2019-nCoV) Nucleocapsid Protein. No cross-reactivity in ELISA with MERS-CoV Nucleoprotein protein, HCoV-229E Nucleoprotein protein, HCoV-NL63 Nucleoprotein protein, HCoV-HKU1, Nucleoprotein protein, HCoV-OC43 Nucleoprotein

Supplier name: Bethyl Laboratories
Catalog number: A80-148B
Antibody validation by vendor: Immunoelectrophoresis and ELISA to confirm that antibody reacts specifically with human IgG and that cross reactivity with IgA and IgM is negligible. No antibody was detected against non-immunoglobulin serum proteins.

Human research participants

Policy information about studies involving human research participants

Population characteristics	All available covariate characteristics were reported in the source data file.
Recruitment	There was no recruitment for any retrospectively collected cohorts (including U. Bonn cohort and all commercial sources) for this study. The CTCH cohort volunteer donors were selected based on symptoms/SARS-CoV-2 PCR status (both positive and negative) and possible exposure to individuals displaying symptoms. The co-resident volunteer cohort was self-selected and offered to the authors by the donors as potentially of interest for this study.
Ethics oversight	The Univ. of Bonn study was approved by the Institutional Review board of the University Hospital Bonn (134/20). All participants in other studies signed written informed consent prior to enrollment; samples were collected under an IRB exemption since these were fully de-identified samples.

Note that full information on the approval of the study protocol must also be provided in the manuscript.
